# Inter-Annual Variability of Fledgling Sex Ratio in King Penguins

**DOI:** 10.1371/journal.pone.0114052

**Published:** 2014-12-10

**Authors:** Célia Bordier, Claire Saraux, Vincent A. Viblanc, Hélène Gachot-Neveu, Magali Beaugey, Yvon Le Maho, Céline Le Bohec

**Affiliations:** 1 Université de Strasbourg, Institut Pluridisciplinaire Hubert Curien, Laboratoire International Associé LIA-647 *BioSensib*, Strasbourg, France; 2 CNRS, UMR-7178, LIA-647 *BioSensib*, Strasbourg, France; 3 AgroParisTech ENGREF, Paris, France; 4 IFREMER – UMR 212– Ecosystème Marin Exploité, Sète, France; 5 Centre d’Ecologie Fonctionnelle et Evolutive, Equipe Ecologie Comportementale, UMR 5175 CNRS, Montpellier, France; 6 Centre Scientifique de Monaco, LIA-647 *BioSensib*, Principality of Monaco; 7 University of Oslo, Centre for Ecological and Evolutionary Synthesis, Department of Biosciences, Blindern, Norway; Norwegian Polar Institute, Norway

## Abstract

As the number of breeding pairs depends on the adult sex ratio in a monogamous species with biparental care, investigating sex-ratio variability in natural populations is essential to understand population dynamics. Using 10 years of data (2000–2009) in a seasonally monogamous seabird, the king penguin (*Aptenodytes patagonicus*), we investigated the annual sex ratio at fledging, and the potential environmental causes for its variation. Over more than 4000 birds, the annual sex ratio at fledging was highly variable (ranging from 44.4% to 58.3% of males), and on average slightly biased towards males (51.6%). Yearly variation in sex-ratio bias was neither related to density within the colony, nor to global or local oceanographic conditions known to affect both the productivity and accessibility of penguin foraging areas. However, rising sea surface temperature coincided with an increase in fledging sex-ratio variability. Fledging sex ratio was also correlated with difference in body condition between male and female fledglings. When more males were produced in a given year, their body condition was higher (and reciprocally), suggesting that parents might adopt a sex-biased allocation strategy depending on yearly environmental conditions and/or that the effect of environmental parameters on chick condition and survival may be sex-dependent. The initial bias in sex ratio observed at the juvenile stage tended to return to 1∶1 equilibrium upon first breeding attempts, as would be expected from Fisher’s classic theory of offspring sex-ratio variation.

## Introduction

In animal populations, determinants of adult sex ratio (ASR, i.e. the proportion of male to female adult individuals [1]) are likely to occur before adulthood, due to variations in parental sexual allocation of resources (before and after birth, reviewed in [2]), and to fluctuations in sex-specific mortality of these offspring before recruitment into the breeding population. According to Fisher [3], parental investment into male and female offspring should be equal at the termination of parental care, as the production of any zygote requires one mother and father. Thus, the reproductive value of males and females should be equal [3] and any tendency to deviate from a 1∶1 sex allocation should return to equilibrium by frequency-dependent selection [4]–[5]. Nonetheless, Hamilton pointed out that biased sex ratios were likely to occur in natural conditions [6], if the costs of producing male and female offspring were different. In such cases, a bias towards the less costly sex should be expected [3], [6]–[8]. In sexually dimorphic species for instance, the higher costs of producing the larger (*e.g.* male) sex should favour a production bias towards the smaller (*e.g.* female) one. Additionally, Trivers & Willard [7] hypothesised that mothers in good condition may afford to invest more into the sex with greatest variance in reproductive success, when mothers in poor condition gain more by investing into the sex less affected by poor maternal rearing conditions [7], [9]. This seems to be the case in the red deer (*Cervus elaphus*) in which high population densities during female pregnancy lead to adverse nutritional stress causing mothers to produce fewer male than female offspring [10]. Alternately, sex-ratio bias may also stem from the fitness benefits/costs associated with inter-sexual differences in competition/cooperation, favouring selection for the sex in which competition between parents and offspring is lowest and/or cooperation highest [6]. In particular, Clark’s resource competition model ([11]; generalisation of Hamilton’s model) highlights an additional cost of producing offspring for mothers, in that they must later compete for food/mate/reproductive site resources. Consequently, mothers might favour the more dispersive sex. For instance, during years of El Niño events, Northern elephant seal (*Mirounga angustirostris*) females experience poor foraging conditions and high nutritional stress [12]. Interestingly, offspring production is biased towards males in those years and this male-biased sex ratio has been suggested as an adaptive strategy to decrease later resource competition between mothers and daughters on similar food patches [13].

Because it has direct consequences on breeder proportions [14]–[15], understanding the dynamics of ASR and the reasons for its fluctuations is an important perspective in animal ecology (e.g. [16], see [17] for a review in birds). In this study, we used a decade-long dataset to investigate sex-ratio variability in a wild seabird population of king penguins (*Aptenodytes patagonicus*). In these seasonally monogamous seabirds, bi-parental care is necessary for successful reproduction, so that a biased ASR should produce adults of the supernumerary sex that are unable to find mates and reproduce, resulting in a reduced number of breeders at the population scale with potential consequences on population dynamics. Surprisingly however, male-biased ASRs have been suggested in king penguin populations [18]–[21] and so has male-biased mate competition [22]. Yet, the reason for such a bias, and life history stage at which it arises, remains unclear. Therefore, using over more than 4000 king penguins of ‘La Baie du Marin’ colony (Crozet Archipelago; Southern Ocean), we considered fledgling sex-ratio variability and its potential links with environmental parameters. The breeding biology of king penguins imposes strong constraints on parental investment. The absence of a nest [23] compels breeding birds to navigate through thousands of conspecifics to locate both partners and chicks [24]. In addition, chick development from hatching to fledging occurs over an exceptionally long period (over 11 months [23], [25]–[26]), encompassing the austral winter when chick growth is limited and mortality is high [26]–[28], chicks gather in crèches to limit predation pressure [29] and increase thermoregulation [30]. This long breeding cycle (over a year from courtship to fledging) also results in non-synchronised laying, early and late offspring being reared in highly heterogeneous conditions (i.e. differences in food resources [31] and bird colony density [32]). In particular, early and late chicks are known to differ in their phenotype at hatching [27] and to exhibit strong differential mortality during the winter [26]–[28]. Finally, as penguins are state-dependent breeders [33], environmental conditions before the onset of breeding might affect parental body condition [34] and foraging efficiency, with potential consequences on chick growth and survival [33]–[34]. Given the above features, king penguins provide an interesting model to investigate how sex ratio may be modulated by environmental factors and whether biased sex ratio may arise over early life history in this species, either due to differential parental allocation or sex-specific mortality. For instance, sex ratio might change between fledging and adulthood, as male and female juveniles may differ in their return rates to their natal colony [35].

## Materials and Methods

### Ethics

Most animals in this study were handled only once (during their first moult) for marking with a subcutaneous transponder tag and measurement of morphological features. A smaller number of individuals had also been previously handled as chicks (in their first month) for marking with a small external plastic pin (Fishtag, Floytag) and blood-sampling. All procedures employed during the field work were approved by the Ethical Committee of the French Polar Institute (Institut Paul Emile Victor – IPEV) and conducted in accordance with its guidelines, also complying with French laws including those relating to conservation and animal welfare. Authorizations to enter the breeding site (permits n° 2005–191 issued on the 21^st^ of November 2005, 2006–67 issued on the 6^th^ of November 2006, 2007–149 on the 24^th^ of October 2007, 2008–98 issued on the 5^th^ of September 2008, 2009–57 issued on the 26^th^ of August 2009, 2010–79 issued on the 3^rd^ of September 2010) and handle birds (permits n° 99/346/AUT issued on the 30th of November 1999, 00/240/AUT issued on the 5^th^ of September 2000, 01/315/AUT issued on the 4^th^ of July 2001, 01/322/AUT issued on the 16^th^ of August 2001, 2003–113 and 2003–114 issued on the 7^th^ of October 2003, 2004–182 and 2004–183 issued on the 14^th^ of December 2004, 2005–203 issued on the 1^st^ of December 2005, 2006–73 issued on the 6^th^ of November 2006, 2007–144 issued on the 24^th^ of October 2007, 2008–71 issued on the 5^th^ of September 2008, 2009–59 issued on the 29^th^ of August 2009, 2010–67 issued on the 3^rd^ of September 2010) were delivered first by the French ‘Ministère de l’Aménagement du Territoire et de l’Environnement’ and then by the ‘Terres Australes et Antarctiques Françaises’ (TAAF). Handled animals were removed from the colony in order to minimize the disturbance to neighbouring birds and carried a few meters away for manipulation. They were hooded to reduce their stress and manipulations lasted between 5 and 10 minutes. The transponder tags weigh 0.8 g and have no known adverse effects. They were shown to have no effect on survival of king penguins [36] or breeding success, recruitment or survival of great tits (*Parus major*) [37]. Furthermore, concerns about infections should be minimal, as transponder tags are kept sealed sterile in iodine capsules (Betadine) and removed from the capsules only by the process of injecting them into the bird. Vétédine soap and alcoholic antiseptic solutions were used to disinfect the skin and the injecting needle before each insertion. Flesh wounds did not appear infected thereafter (personal observations on a sample of recaptured birds). Blood was sampled in small quantities, taking into account the age of the chick (1 mL for fledglings and 100 µL for younger chicks).

### Long-term monitoring and sex-ratio determination

Our study was conducted in the king penguin colony of ‘La Baie du Marin’, on Possession Island, Crozet Archipelago (46°25′S, 51°45′E). From 2000 to 2009, we marked 3787 king penguin chicks that we later monitored using an automatic system (ANTAVIA), based on radio-frequency identification (RFID) of individuals born in a subpart of the colony [38]. Around the peak of fledging, chicks in advanced moult were captured on the edge of the colony and implanted with passive transponder tags under the skin of their leg, without any other external mark. Upon capture, the chick’s head was covered with a hood to keep it calm. A 1-mL blood sample was collected from its marginal flipper and used for sex determination based on genomic DNA (see below). Because those chicks were initially captured right before fledging, we did not know whether they were issued from an early (laying prior to Jan. 1^st^) or late reproduction [23], [26]. Thus, we captured and marked 200 early and 200 late chicks per year from 2007 to 2009 (∼3–4 week old). Those chicks were temporarily tagged with a small external plastic pin (Fishtag, Floytag). Of those, 225 chicks (198 early chicks and 27 late chicks) survived to fledging and were re-captured, transponder-tagged and blood-sampled at that time. Finally, as part of a separate study, 20 early and 20 late chicks were captured in 2009, and 173 early and 197 late chicks were captured in 2010, right after hatching (∼1 week old). Those were marked with a fishtag, blood sampled (∼100 µL), and used to investigate the changes in sex ratio between hatching and fledging.

Chick sex was determined from DNA extracted from blood samples following the protocol described by Sambrook et al. [39]. Sex determination relied on polymerase chain reaction (PCR) amplification of two parts of the sex chromosome CHD1 gene, which in birds differs in size between the Z and W chromosomes [40], [41]. Females were characterized by displaying both a W-specific fragment and a Z-specific fragment, while males showed only the shorter Z-fragment. All individuals were tested with primers F1/R1 [42], and in each year, ca. 10% of them were controlled at random using another pair of primers P2/P8 [43] to confirm sex determinations. Sex ratio was calculated as the proportion of males in the population (given as a %).

### Chick morphological measurements and body condition

Standardised measurements of flipper length, beak length (±1 mm) and body mass were collected on all but 3 chicks upon capture right before fledging (N = 4101). Flipper and beak lengths are highly repeatable measurements [44] and provide good descriptors of structural size. However, because those measurements are correlated (Spearman’s rank correlation; *p*<0.001, *rho* = 0.32, *N* = 4101), we established a Structural Size Index (SSI) using principal component analysis as previously described in [35]. The first principal component of the analyses explained 79% of the variation in bill and flipper length and was retained as the SSI 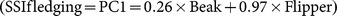
_._

Because body mass is highly variable in king penguins and perhaps associated with differences in nutritional status as well as structural size, OLS-regression residuals of body mass on structural size are likely to provide a better reflection of the actual energy stores of the animal [45]. Thus, we calculated body condition as the residuals of an OLS-regression of chicks’ body mass on SSI [35], [46].

To determine if the sexual size dimorphism occurred at hatching or fledging, we also calculated body condition from an OLS regression of body mass on structural size at hatching over 173 early and 197 late-hatched king penguin chicks in 2010 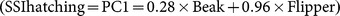
_._

### Life-history determination of transponder-tagged birds

Once implanted with a transponder tag, chicks are automatically identified throughout their lifetime in the ANTAVIA sub-colony by RFID antennas. Those antennas are buried underground at unique transit pathways in and out of the sub-colony and allow continuous monitoring of bird movements and presence within the colony [38]. Such data have yielded detailed information on individuals’ lives after fledging, including data on first return rates to their natal colony [35], reproduction [47]–[49], age-specific survival and breeding performances (Le Bohec et al., *unpublished data*). Thus, monitoring individuals tagged from fledging allows assessment of changes in sex ratio throughout different life-history stages. This information was used to investigate changes in sex ratio between fledging, first return to the colony, and first reproductive attempt. For this analysis, only the first 4 cohorts of tagged chicks were used, as most of the birds from latter cohorts had not yet started to breed.

### Environmental descriptors

#### Climate and oceanographic conditions

To investigate how sex ratio may be influenced by climatic conditions, we used indicators of processes acting both at local and global scales [50]–[51]. A large-scale climatic index, the Southern Oscillation Index (SOI; calculated on a monthly basis as the fluctuation in the air pressure difference between Tahiti and Darwin) was obtained from the Australian Bureau of Meteorology. Prolonged periods of negative SOI values are usually associated with warm ocean temperatures in our study area, typical of El Niño events [52]. We used Sea Surface Temperature (SST, in °C), as a local proxy of prey abundance and distribution, a good indicator for marine predators such as king penguins [35], [53]–[55]. Indeed, by affecting primary production, SST has strong consequences on prey abundance [56]. Monthly SST values were obtained from the National Ocean and Atmospheric Administration of the USA and averaged for the feeding areas (46–60°S, 46–56°E) of king penguins during the summer (Polar Front, PF) and the winter (Marginal Ice Zone, MIZ) [57]. We previously found that breeding success in our study colony was mostly affected by the SOI and SST conditions of the same year [53]. However, we tested for SOI and SST effects over four different periods: 6- and 12-mo time-lag prior to laying (prior to the mean annual laying date of early breeders, May–Oct. or Nov.–Oct.), 6-mo time-lag after laying (after the mean annual laying date of early breeders, Nov.–Apr.), and over the entire breeding cycle (from laying to fledging, Nov.–Oct.). The different time-periods yielded similar results, so that results are given only over one period: the summer season, i.e. the 6-mo period following the onset of reproduction (the mean annual laying date of early breeders). Finally, we recorded the mean annual laying date of early breeders from 2004 to 2009 as a proxy of environmental conditions before breeding onset, and calculated the mean annual breeding success of the colony from 2000 to 2009 (Le Bohec et al., *unpublished data*) as a proxy of the annual environmental conditions endured during reproduction. Early years and/or years of high breeding success could be viewed as more favourable years of high resource availability.

#### Colonial breeding conditions

Because colony density of king penguins varies to a large extent over the course of a breeding season and may affect the physiological status of breeding parents [32], we considered whether colony density might affect chick sex ratio. Each year, starting in 2004, we calculated yearly colony density indices in the ANTAVIA sub-colony by counting breeding individuals on pictures taken in December (peak density of the breeding season). We further calculated a second annual density index as the proportion of transponder-tagged birds monitored as breeding in a given year among tagged birds alive and of breeding age.

### Statistics

**Figure 1:**
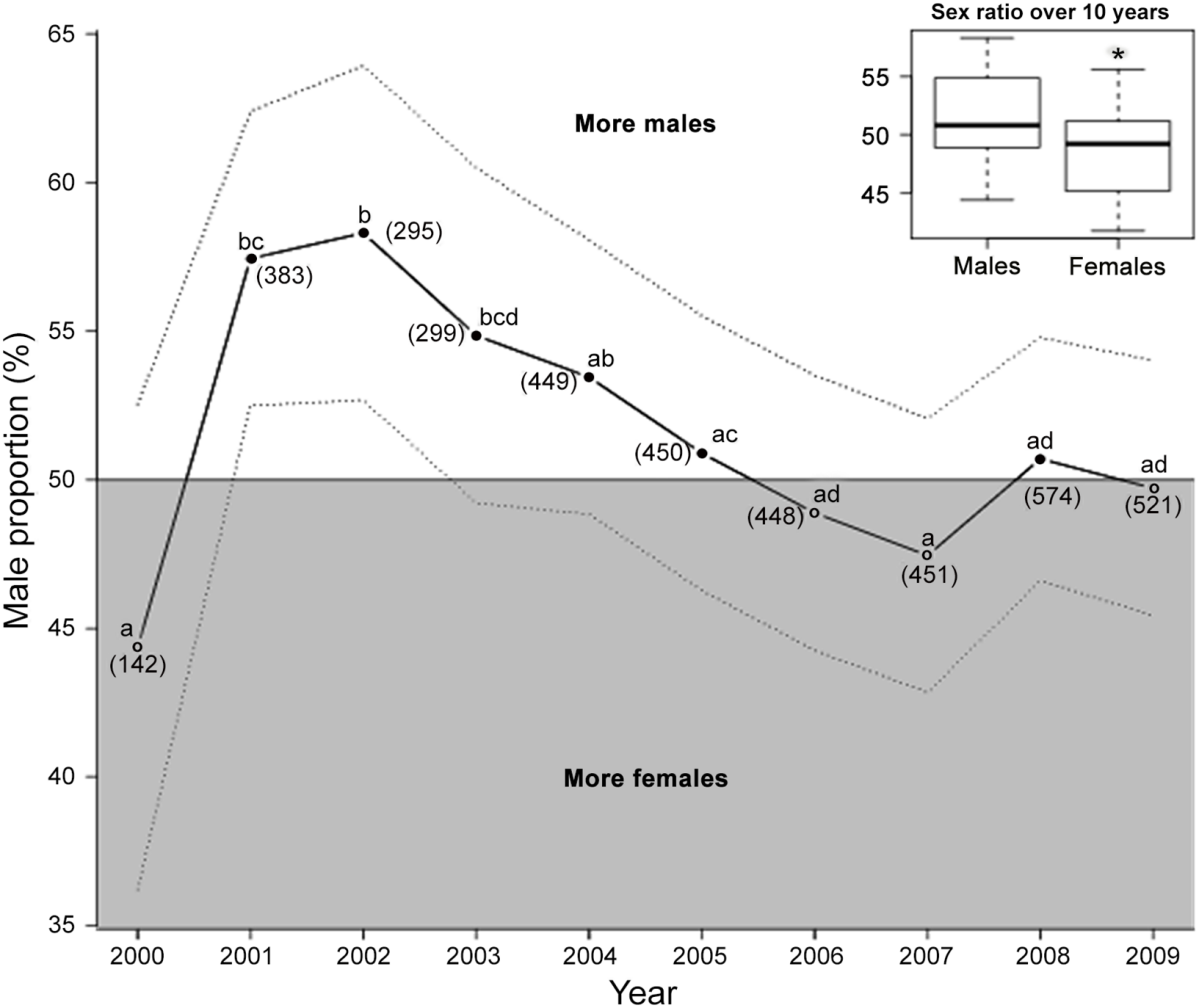
Sex ratio of fledging king penguins over a 10-year period. Sex ratio is presented as male proportion (%). Dotted lines represent the 95% confidence interval. The grey area of the figure (fledging males <50%) corresponds to female-biased sex ratios, while the white area corresponds to male-biased sex ratios. Points not sharing a common superscript are different for *P*<0.05. Sample sizes are given in brackets. The insert shows the average sex ratio over the 10-year study (*significant difference for *P*<0.05).

All statistics were run using R v. 2.13.0 [58]. Because chicks were randomly sampled in the absence of their parents, we could not know whether sampled chicks may come from the same breeding pairs from year to year. This prevented us from looking for pseudo-replication in our data and using Generalized Linear Mixed Models (GLMMs) that would take adult identity into account. Yet, it should be noted that no more than 500–600 chicks were marked per year in a part of the colony retaining *ca.* 8,000 breeding pairs, so that the probability of pseudo-replication should be low. Therefore, Generalized Linear Models (GLMs; maximum likelihood approach) fitted to a binomial distribution were used to evaluate sex ratio with environmental factors or laying period. As all independent variables (*e.g.* laying period) were not available for all individuals, we could not run a single general model and use an information theoretical approach to select variables. Instead, we had to compute separate models for different independent variables. Still, in the case of climatic variables, we ran a single global model with SST and SOI as independent variables. The most appropriate models were selected using Akaike’s Information Criterion (AIC). Adjusted R^2^ values are indicated along with p-values. Data were checked for normality and for between-group homoscedasticity and between-year comparisons were performed using Wilcoxon rank-sum tests. Sex-ratio comparisons across life-history stages were performed using a binomial proportion test ('prop.test' package in R). Variables were considered significant for *P*<0.05. Bonferroni corrections were considered for multiple testing in three occasions, i.e. comparisons of sex ratio between life-history stages 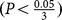 comparisons of body condition between males and females for each year 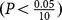, and finally pairwise comparisons of sex ratio between years 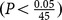. In the first 2 cases, results were similar regardless of whether the Bonferroni adjustment was applied or not, indicating their robustness. In the last case (pairwise comparison of annual sex ratios), differences appeared. As Bonferroni adjustments are often criticized for being too conservative preventing from detecting significant differences, we decided to present the results without this adjustment on Figure 1. The number of birds is given as *N*.

## Results

### Sex ratio at fledging and inter-annual variability

Over the 10-year study period, fledging sex ratio was slightly biased towards males (1941 females *vs.* 2071 males), implying that an overall greater proportion of male offspring successfully fledged from 2000 to 2009, *i.e.* 51.62% of male (GLM; *z* = 2.05, *P* = 0.04, *N* = 4012; upper-right panel, Fig. 1). However, fledging sex ratio varied significantly between years (Pairwise tests given in Fig. 1) and significant male-bias was observed in only 2 of the study years (GLM; *z* = 2.90, *P* = 0.004, *N* = 383 and *z* = 2.84, *P* = 0.004, *N* = 295 for 2001 and 2002, respectively; Fig. 1). Because the data were limited to 10 points, we could not perform breakpoint analyses, but from a visual inspection (Fig. 1) it seems that the proportion of fledged males increased from 2000 to 2002 (44.37% to 58.31%, *z* = 2.39, *P* = 0.02, *N* = 820), before decreasing continuously over 2002–2007, from 58.31% to 47.45% (*z* = −3.39, *P*<0.001, *N* = 2392). Finally, sex ratio at fledging was stable over the last 3 years (2007–2009) of the study (*z* = 0.70, *P* = 0.48, *N* = 1546).

### Sex ratio and breeding timing: Early *vs.* late-hatched chicks

**Figure 2:**
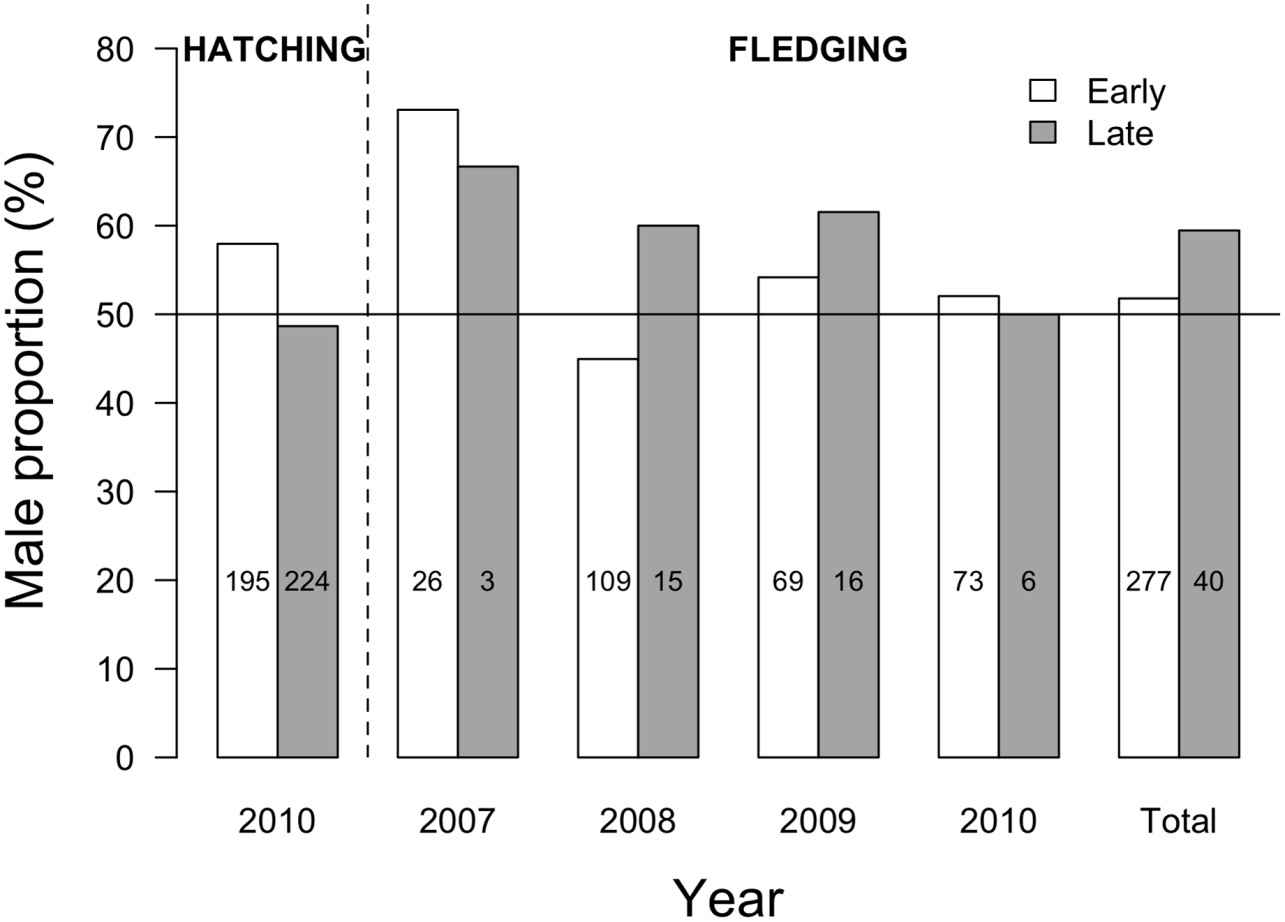
Sex ratio of king penguin chicks produced either early or late during the 2007–2010 breeding seasons. Sex ratio is presented as male proportion (%). White and grey bars correspond to early and late chicks, respectively. In 2010, chick sex was determined both at hatching and fledging. Sample sizes are specified in the bars.

In 2010, the proportion of hatched males tended to be greater for early (57.95%) than for late (48.66%) chicks (GLM; *z* = −1.90, *P* = 0.06, *N* = 419; Fig. 2). Also in 2010, we found that chick sex ratio did not vary between hatching and fledging (which included the austral winter period), both in early (–5.90%; Proportion test; *P* = 0.47, *N* = 195/73) and late (+1.34%; *P* = 1, *N* = 224/6) chicks (see Fig. 2).

From 2007 to 2010, we determined fledgling sex ratio for chicks hatched either early or late in the season (Fig. 2). Although the overall proportion of males appeared higher in late chicks (62.50% *vs.* 52.35% for early chicks), this difference was not statistically significant over the years (GLM; *z* = 0.88, *P* = 0.38, *N* = 317). Similarly, we observed no significant difference in early- *vs*. late-chick sex ratio when years were considered separately (GLM_2007_; *z* = 0.23, *P* = 0.81, *N* = 29; GLM_2008_; *z* = 1.08, *P* = 0.28, *N* = 124; GLM_2009_; *z* = 0.49, *P* = 0.62, *N* = 85; GLM_2010_; *z* = −0.10, *P* = 0.92, *N* = 79).

### Sex ratio and life-history stages

Sex ratio at chick-fledging (55.22%), when juveniles first return to the colony (54.62%), or at first breeding attempt (53.27%) did not vary among cohorts hatched between 2000 and 2003. Sex ratio did not change significantly between fledging and first returns (Proportion test; *P* = 0.83, *N* = 1112/866), between first returns and first breeding attempts (*P* = 0.65, *N* = 866/597), or between fledging and first breeding attempts (*P* = 0.47, *N* = 1112/597).

### Fledging sex ratio and environmental factors

#### Climatic parameters

Over the 10-year study (2000–2009) and 4012 individuals, the most appropriate models, selected by AIC, did not show any effect of SOI or SST on fledgling sex ratio. Indeed, the null model was retained regardless of the tested period (before breeding, summer after laying, and entire breeding cycle).

**Figure 3:**
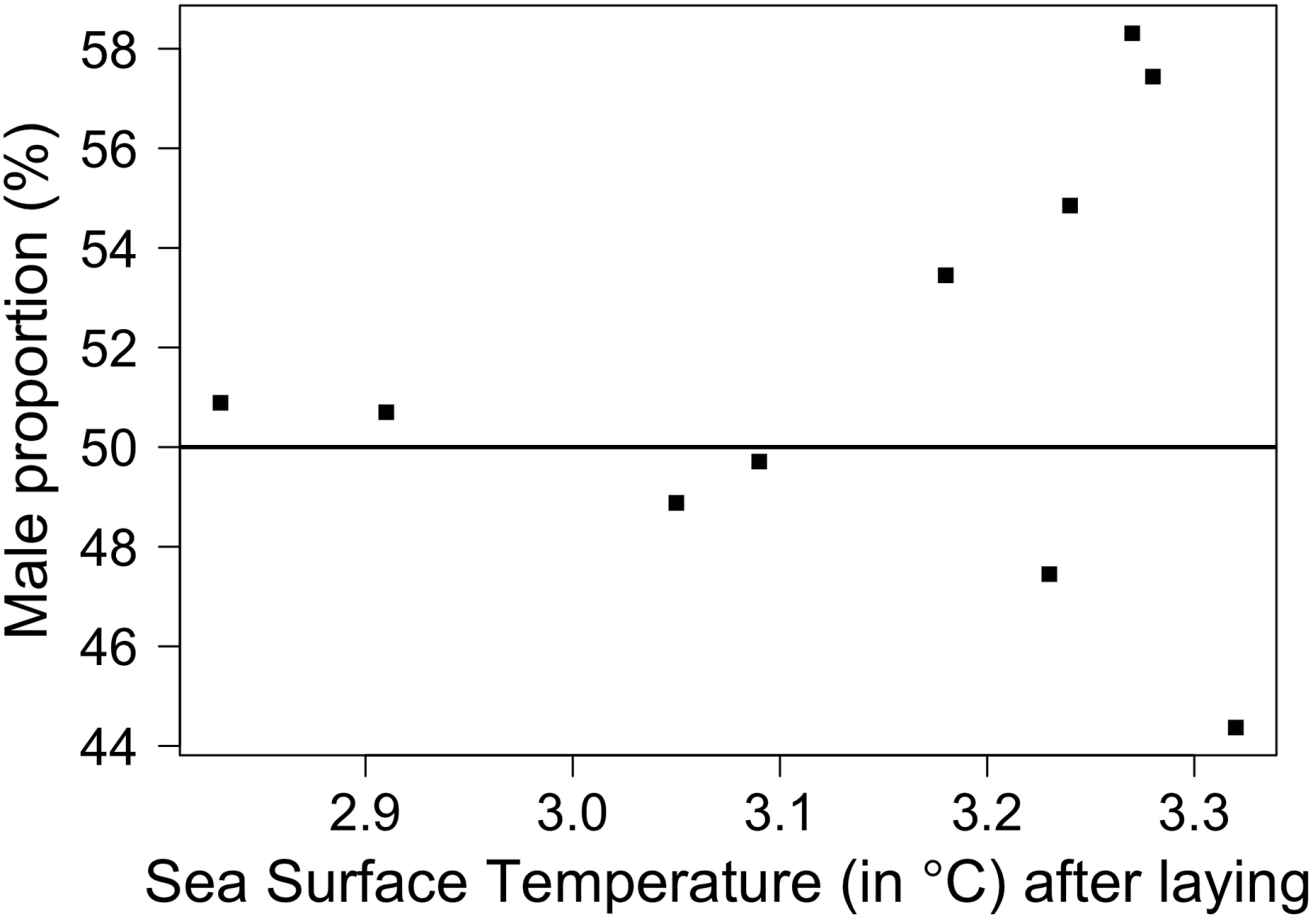
Changes in sex ratio of fledging king penguins according to the Sea Surface Temperature (SST) over a 10-year period. Sex ratio is presented as male proportion (%). SST (°C) was averaged over the summer season, *i.e.* the 6-mo period following the onset of reproduction (mean annual laying date of early breeders) (see methods).

However, it is interesting to note that the period of decreasing male proportion at fledging (2002–2007, except in 2006) coincided with negative values of SOI during the period of incubation and chick-brooding. Also, variability in fledging sex ratio tended to increase with SST (Fig. 3), i.e. warmer years produced very unbalanced sex ratios (whether in favour of males or females) compared to colder years during which the sex ratio was close to 1∶1.

From 2004 to 2009, the mean annual laying date of early breeders did not affect annual sex ratio at fledging (GLM; *z* = −0.02, *P* = 0.99). Similarly, the mean annual breeding success of the colony over the 10-year study (2000–2009) was not related to fledgling sex ratio (GLM; *z* = 1.07, *P* = 0.28).

#### Colonial environment

From 2004 to 2009, sex ratio at fledging was not related to colony density (global colony-counts of individual penguins; GLM; z = 0.76, *P* = 0.44). Colony density as estimated only by counting tagged birds reproducing in the colony from 2007 to 2009, yielded similar results (GLM; *z* = 0.94, *P* = 0.35).

### Fledging sex ratio and chicks’ structural size and body condition

According to the structural size index, no dimorphism was observed at hatching neither for the 2010 early-hatched king penguin chicks (Wilcoxon; *W* = 4378, *P* = 0.86, *N* = 173), nor for the 2010 late-hatched chicks (Wilcoxon; *W* = 4378, *P* = 0.24, *N* = 197).

**Figure 4:**
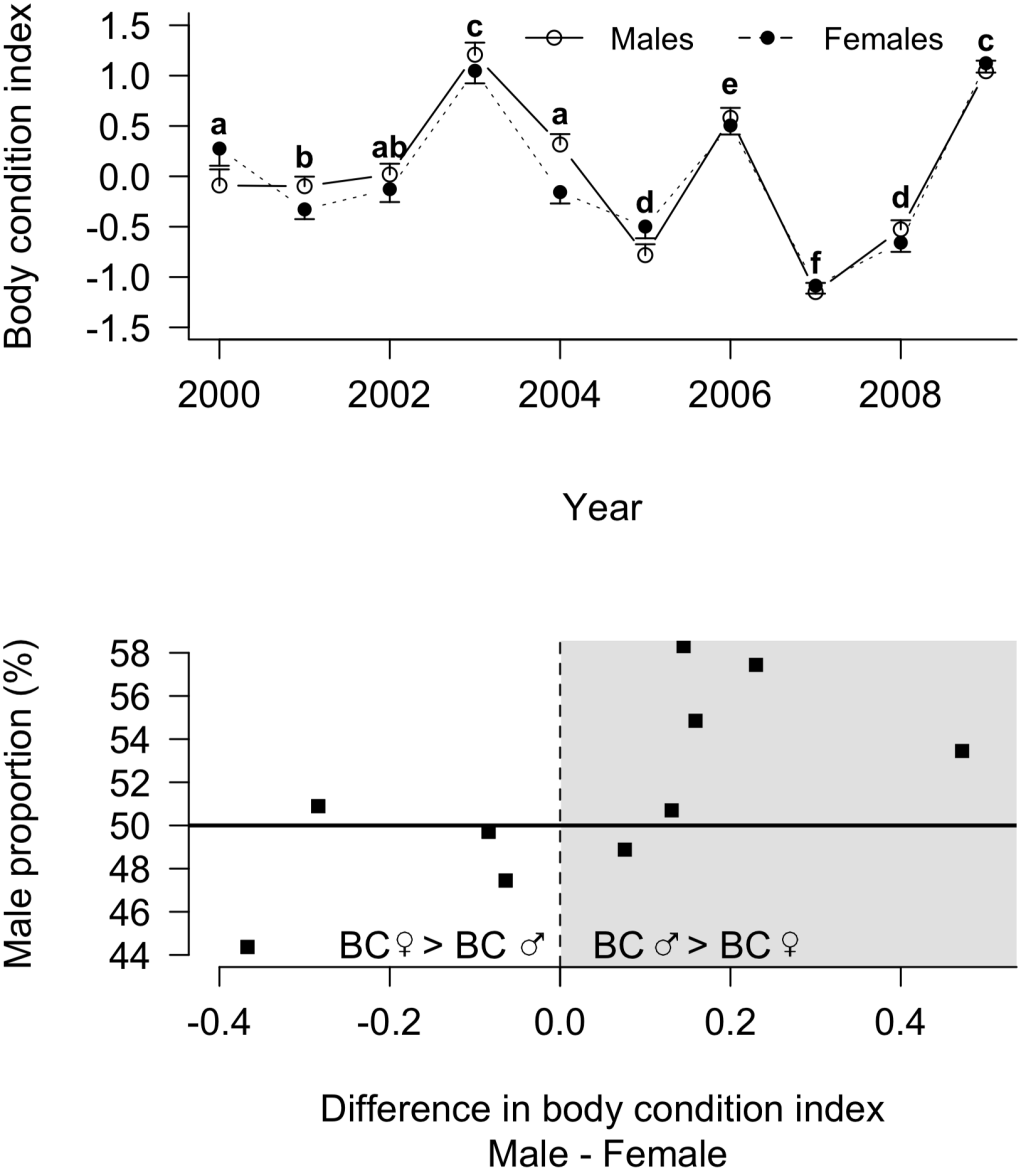
Yearly changes in male and female fledging body condition (BC; panel A) and yearly sex ratio at fledging according to fledging BC difference between males and females (panel B) in king penguins. Panel A: male and female body conditions are given as solid line, open circles and dotted line, full circles, respectively. Letters in superscript indicate differences between years in the average (male and female) body condition. Average values not sharing a common superscript are different for *P*<0.05. Panel B: the grey area corresponds to the years in which male body condition is higher than female body condition, while the white area presents the opposite. Note that the horizontal solid line indicates the balanced sex ratio at fledging. Every point situated above corresponds to a higher proportion of males, while below corresponds to a higher proportion of females.

At fledging, however, chicks exhibited sexual size dimorphism, males being larger than females according to the structural size index (3.36±0.26 *vs.* −3.59±0.25, Wilcoxon; *W* = 1372092, *P*<0.001, *N* = 4101). However, when looking at body condition at fledging, the difference between males and females was not constantly in favour of males (Fig. 4A). Males had a higher body condition than females only in 2004 (Wilcoxon; *W* = 21025, *P* = 0.003, *N* = 449).

The yearly difference in body condition between males and females was correlated with sex ratio at fledging (*r* = 0.67, *P* = 0.04; Fig. 4B). Higher male proportions were associated with a higher male body condition when compared to females.

## Discussion

Over a 10-year period, fledgling sex ratio in our king penguin population was slightly male-biased. This finding is in line with previous data on male-biased ASR both in the present [18], [48] and other king penguin colonies (on Kerguelen [19]–[20] and South Georgia [21] archipelagos). Interestingly, biased ASR in birds appears to mainly result from adult sex-specific mortality, rather than biased offspring sex ratios. Indeed, in a review of 140 estimates of offspring sex ratio, only few (17%) differed significantly from 1∶1 (9.3% female biased and 7.8% male biased) [59]. Yet, despite slightly higher annual survival on average for males *vs.* females (94.2% *vs.* 90.7%), this difference is not significant ([48] and unpublished results over 1999–2010). Our result on biased sex ratio at fledging is thus relatively unusual for birds. Yet, it should be noted that despite a high sample size, the bias was only barely significant, indicating that fledgling sex ratio was mainly maintained close to the Fisher equilibrium of 1∶1. In agreement with previous studies in adults [25], [44], we found that king penguin fledglings also exhibited slight sexual dimorphism in favour of males, probably a result of differential growth, as no sexual dimorphism was observed at hatching for king penguin chicks (though size at hatching was available only for one cohort). Higher costs of raising males might therefore be expected. Given the equalization of fitness of male and female offspring at the termination of parental care, theory would then predict an even or slightly female-biased sex ratio at fledging [3]. Of course, for this to be true, costs of raising male and female offspring actually need to differ and remain to be accurately measured (*e.g.* in terms of energy allocation). Nonetheless, our results might thus appear in this regard to contradict Fisher’s theory because a significant male-bias occurred [3]. However, this is perhaps not surprising given that life histories of birds clearly violate several assumptions of the standard sex allocation models (*e.g.* extended biparental care, overlapping generations, see [60] for a review).

In contrast to the overall limited sex-ratio bias, we observed high interannual variability in fledging sex ratio ranging from 44% to 58% of males. Such fluctuation at fledging may result from yearly fluctuations in primary sex ratio and/or from differential chick survival between hatching and fledging. Unfortunately, we only had one year of data on hatching sex ratio, so that our findings have to be considered preliminary. Nonetheless, they suggest that sex ratio could be biased as soon as hatching, but also that sex-biased chick mortality may occur between hatching and fledging. Finally, we found that biased sex ratios at fledging were associated with biased body conditions in male and female fledglings. When sex ratio was male-biased, males presented a higher body condition than females, and reciprocally. Because fledgling body condition affects post-fledging return rates and may be viewed as an index of chick quality [35], this may suggest that parents adopt sex-specific production strategies depending on yearly environmental conditions. For instance, larger males may be more vulnerable than females to food shortage [61] in specific years, making it a better option to produce females under harsh environmental conditions [7]. Also, parents in poor condition (e.g. nutritional stress or lack of experience) may favour the less costly sex either to avoid impairing future fitness or because reproduction is likely to fail, *i.e.* the ‘cost of reproduction hypothesis’ [62]–[65]. Alternatively, females may survive better than males in poor years, even under equal investment, because of lesser energy requirements during the growth period.

In our study, the proportion of hatching males tended to be higher early in the season (58 *vs.* 49% late in the season), although our small sample size of late fledglings prevents us from definite conclusions. Such seasonal variability in sex ratio has been previously reported in the painted turtle (*Chrysemys picta*) [66], and may result from poor foraging and nutritional conditions, because of mismatch with peak resource productivity (*e.g.* [67]–[68]). In king penguins, late breeding birds spend longer foraging trips at sea during chick-rearing and build up larger fuel reserves than early birds, reflecting the well-known trade-off between adult maintenance and offspring care/survival in long-lived birds [31], [62]. If male chicks indeed require larger amounts of food than females, a change in parental provisioning late in the season may affect male mortality, thus explaining the observed female bias in sex ratio.

Fledging sex ratio was relatively insensitive to environmental descriptors measured in this study. Yearly population (colony) density did not affect fledging sex ratio. Although effects of population density on sex ratio have been reported in mammals [10], those effects were mediated by maternal condition affected by high competition on nutritional resources [10]. However, intra-specific competition may be less of a problem for king penguins, for which foraging success appears to be more influenced by local oceanic characteristics of food patches than by density-dependent processes (see [69] for a discussion). Similarly, global climatic conditions (SOI) did not appear to have an effect on fledging sex ratio. However, whether this is a result of sex ratio truly being insensitive to global oceanographic conditions or because only relatively mild La Niña/El-Niño events occurred over the 10-year monitoring period (low amplitude in annual SOI variation, see [70]) remains to be examined. Finally, no effect of yearly local environmental conditions (SST) on fledging sex ratio was highlighted in this study. Although we did record a significant bias in fledging sex-ratio variability with increasing SST (cold years were associated with sex ratios close to 1∶1, whereas warm years resulted in highly biased sex ratios), this bias was not consistently in favour of either sex, and our large sample sizes suggest that yearly changes in sex ratio were likely just related to stochasticity in environmental parameters (including SST) affecting chick mortality.

Finally, if understanding which parental/environmental variables influence offspring sex ratio is fundamental to the breeding and evolutionary biology of king penguins, one should bear in mind that it is actually the ASR that is a key parameter to population dynamics. In our study colony, although the sex-ratio bias was 2 percentage points lower at recruitment (*i.e.* first breeding attempt) than at fledging (suggesting a return towards equilibrium at recruitment), this decrease was not statistically significant.

To conclude, our results over a 10-year period hint to potential effects of oceanographic conditions on fledging sex ratio. With a predicted increase in warm climate episodes (IPCC 2013, [71]), investigating king penguin sex ratio over later life-history stages appears the next step to assess its importance in modulating population trajectories and population persistence over time [72]. From results to date, it appears that an expectation of considerable fluctuations in fledging sex ratio might accompany further ocean warming.
